# Systems analysis and improvement to optimize opioid use disorder care quality and continuity for patients exiting jail (SAIA-MOUD)

**DOI:** 10.1186/s13012-024-01409-0

**Published:** 2024-12-18

**Authors:** Sarah Gimbel, Anirban Basu, Emily Callen, Abraham D. Flaxman, Omeid Heidari, Julia E. Hood, Anna Kellogg, Eli Kern, Judith I. Tsui, Ericka Turley, Kenneth Sherr

**Affiliations:** 1 Department of Child, Family and Population Health Nursing, University of Washington School of Nursing, 1959 NE Pacific St, Seattle, WA 98195 USA; 2 Department of Global Health, University of Washington Schools of Medicine and Public Health, 3980 15th Ave NE, Seattle, WA 98105 USA; 3 CHOICE Institute, University of Washington School of Pharmacy, 1959 NE Pacific St, Seattle, WA 98195 USA; 4 Department of Health Metrics, Institute for Health Metrics and Evaluation, University of Washington, 3980 15th Ave NE, Seattle, WA 98105 USA; 5 Public Health – Seattle & King County, Chinook Building, 401 5th Ave Ste 1250, Seattle, WA 98104 USA; 6 Department of Epidemiology, University of Washington School of Public Health, 3980 15th Ave NE, Seattle, WA 98105 USA; 7 Department of Health Systems and Population Health, University of Washington School of Public Health, 3980 15th Ave NE, Seattle, WA 98105 USA; 8 Department of Medicine, University of Washington Harborview Medical Center, 325 9th Ave, Seattle, WA 98104 USA; 9 Jail Health Services, Public Health – Seattle & King County, 401 5th Ave Ste 1000, Seattle, WA 98104 USA; 10 Department of Industrial & Systems Engineering, University of Washington, 3900 E Stevens Way NE, Seattle, WA 98195 USA

**Keywords:** Systems analysis and improvement approach (SAIA),, MOUD, Buprenorphine, CFIR, ORIC, Process mapping, Cascade analysis, Continuous quality improvement, Implementation science, Systems engineering, Jail, Overdose, Linkage to care

## Abstract

**Background:**

Between 2012–2022 opioid-related overdose deaths in the United States, including Washington State, have risen dramatically. Opioid use disorder (OUD) is a complex, chronic, and criminalized illness with biological, environmental, and social causes. One-fifth of people with OUD have recent criminal-legal system involvement; > 50% pass through WA jails annually.

Medications for Opioid Use Disorder (MOUD) can effectively treat OUD. WA has prioritized improving access to MOUD, including for those in jails. As patients in jail settings are systematically marginalized due to incarceration, it is critical to foster connections to MOUD services upon release, an acknowledged period of high overdose risk. Currently, there is insufficient focus on developing strategies to foster linkages between jail-based MOUD and referral services.

The *Systems Analysis and Improvement Approach (SAIA)*, an evidence-based implementation strategy, may optimize complex care cascades like MOUD provision and improve linkages between jail- and community-based providers. SAIA bundles systems engineering tools into an iterative process to guide care teams to visualize cascade drop-offs and prioritize steps for improvement; identify modifiable organization-level bottlenecks; and propose, implement, and evaluate modifications to overall cascade performance. The SAIA-MOUD study aims to strengthen the quality and continuity of MOUD care across jail and referral clinics in King County, WA, and ultimately reduce recidivism and mortality.

**Methods:**

We will conduct a quasi-experimental evaluation of SAIA effectiveness on improving MOUD care cascade quality and continuity for patients receiving care in jail and exiting to referral clinics; examine determinants of SAIA-MOUD adoption, implementation, and sustainment; and determine SAIA-MOUD’s cost and cost-effectiveness. Clinic teams with study team support will deliver the SAIA-MOUD intervention at the jail-based MOUD program and three referral clinics over a two-year intensive phase, followed by a one-year sustainment phase where SAIA implementation will be led by King County Jail MOUD staff without study support to enable pragmatic evaluation of sustained implementation.

**Discussion:**

SAIA packages user-friendly systems engineering tools to guide decision-making by front-line care providers to identify low-cost, contextually appropriate health care improvement strategies. By integrating SAIA into MOUD care provision in jail and linked services, this pragmatic trial is designed to test a model for national scale-up.

**Trial registration:**

ClinicalTrials.gov NCT06593353 (registered 09/06/2024; https://register.clinicaltrials.gov/prs/beta/studies/S000EVJR00000029/recordSummary).

**Supplementary Information:**

The online version contains supplementary material available at 10.1186/s13012-024-01409-0.

Contributions to the literature

- Our study examines whether the introduction of an evidence-based, user-friendly, low-cost package of systems engineering tools can optimize investments in providing medications for opioid use disorder for individuals during the high-risk period immediately after exiting jail.
- This is the first application of the Systems Analysis and Improvement Approach (SAIA) across health system levels to optimize both care delivery and continuity of care.
- Our study is designed to provide decision-makers with evidence they need to decide whether to invest in systems engineering approaches during the expansion of MOUD in jail settings, including information on acceptability, feasibility, effectiveness and cost.

## Background

Over the last decade opioid overdose deaths have increased across the United States, including in Washington State (WA), where overdose accounted for 36% of all injury deaths in 2022 [1]. In King County, the most populous county in WA, opioid-related deaths doubled between 2020 and 2023 [2]. Overdose, especially related to opioids, is the leading cause of death for people who have been incarcerated [3, 4]. Immediately after release from carceral settings, individuals with OUD are at greatly increased risk for fatal opioid overdose [5, 6]. Structural racism compounds harms related to opioid use and incarceration, as generations of racist policies and practices disproportionately criminalize communities of color and social determinants of health impede access to opioid use disorder (OUD) prevention, harm reduction, and treatment [7–9]. As a result, Black, Hispanic, American Indian, and Alaska Native communities have experienced sharper increases in opioid overdose deaths, compared to white communities [10, 11].

Treatment with evidence-based agonist medications for opioid use disorder (MOUD), including buprenorphine and methadone, are effective for preventing overdose, [12–14] managing OUD, [15] and preventing recidivism [16]. The steps required to manage OUD are collectively known as the MOUD cascade of care and include living with undiagnosed OUD, OUD diagnosis, engagement in care, initiation of MOUD, long-term retention in care, and remission [17]. Despite common misconceptions that people who use drugs are not interested in clinical management of OUD, many patients using opioids want to quit or reduce their intake. A 2019 survey of WA residents utilizing syringe service programs showed that, of patients who mainly use heroin, 82% wanted to reduce or stop use and 70% were interested in MOUD [18]. With increasing recognition of the importance of social determinants of health like secure housing, adequate income, and food security on health outcomes, evidence suggests that patients on MOUD may have better outcomes with greater integration of clinical treatment and interventions targeting the social determinants of health [19]. This is especially relevant among individuals exposed to incarceration and at heightened risk for overdose. Settings such as jails must therefore engage people in a MOUD care cascade tailored to their setting, [20] including steps such as providing options for treatment with MOUD, administering MOUD, providing connections to recovery-supportive social services and connecting patients to clinical referral sites upon release. Completion of all cascade steps is necessary to reduce incarcerated patients’ risk of overdose and break cycles of incarceration.

There is a critical gap in knowledge surrounding implementation strategies to improve adoption, equity, and sustainment of MOUD care cascade services in jails and linked referral clinics in the community. There is an unprecedented influx of resources in WA and nationally being directed toward implementing MOUD, referring patients on MOUD to other services during care coordination visits, and ensuring that patients are connected to MOUD upon release [21]. However, there are limited resources and attention targeting development of evidence-based implementation strategies to optimize the MOUD care cascade between carceral settings and referral clinics upon release. Carceral settings are harmful to health and often not structurally aligned with the provision of respectful healthcare [22, 23]. Thus, implementing therapeutic interventions such as MOUD in jails requires tailored strategies to address challenges that are unique to these settings. To most effectively reduce opioid overdose risk—among other harms related to criminal legal system involvement [23]—the use of incarceration should be reduced or eliminated. It is therefore critical to focus improvements on linkages to post-release services to align with public health strategies to divest from OUD criminalization. Implementation strategies that address systemic weaknesses in MOUD services between carceral settings and referral clinics are therefore urgently needed.

Evidence-based systems-level implementation strategies like the *Systems Analysis and Improvement Approach* (SAIA) [24–26] improve care cascade efficiency, communication and accountability between providers, promote consensus decision-making in complex systems, and are potentially scalable across public health systems [27–34]. Though routine data exist to guide improvement efforts across varied clinical cascades, frontline providers are rarely engaged in the use of data to guide facility-level decision-making. Systems engineering methods utilize this data and systems thinking to 1) identify the main drivers for inefficiency across care cascades, 2) support provider decision-making to prioritize interventions, and 3) improve integration of services to meet the diverse clinical needs of patients [25, 28, 35]. SAIA bundles systems engineering tools into an iterative implementation strategy [35] designed to optimize multi-step complex cascades, such as the MOUD cascade across the jail-to-referral clinic continuum [25]. SAIA may be a vital strategy to address critical weaknesses in MOUD delivery systems for marginalized patients in King County, WA, and nationally.

SAIA-MOUD aims to make healthcare services more efficient and equitable across jail-based MOUD programs and clinical referral sites so that OUD care for patients leaving jail will be more continuous, equitable, and of higher quality. This may improve linkages to MOUD referral clinics and retention in care following release, ultimately improving OUD-related health outcomes, reducing criminal legal system involvement, and preventing overdose mortality. By focusing improvement efforts on the critical linkages between carceral settings and MOUD referral clinics, SAIA has the potential to create more responsive systems at a vulnerable moment in patients’ social context. Realizing the aims of SAIA-MOUD may create a scalable model for strengthening MOUD care between jail and referral clinics.

## Goals and objectives

The overall goal of this study is to strengthen the quality and continuity of MOUD care across jail and community settings in King County, WA by applying and evaluating a multi-component implementation strategy – the SAIA. Our population of interest are individuals who take MOUD during incarceration in King County, WA jails (JHS) and are subsequently referred to MOUD clinics in the community upon release. We hypothesize that organizations exposed to the SAIA will actively identify modifiable barriers to completing steps in the MOUD cascade and apply locally defined innovations that will lead to measurable improvements in the care and continuity of MOUD services for their patients over and above those observed in patients referred to MOUD clinics not exposed to the SAIA intervention. Over the five-year project, we will conduct a quasi-experimental evaluation of the effectiveness of SAIA on improving MOUD care cascade quality and continuity for patients receiving care in jail and exiting to referral clinics (Aim 1); examine determinants of SAIA-MOUD adoption, implementation, and sustainment (Aim 2); and determine SAIA-MOUD’s cost and cost-effectiveness (Aim 3).

## Methods

### Description of the SAIA-MOUD implementation strategy

**Fig. 1:**
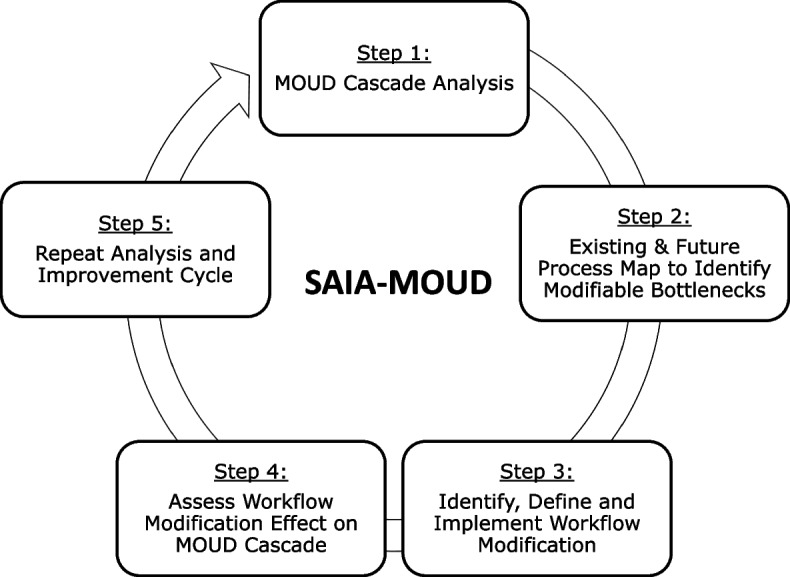
SAIA-MOUD overview

The SAIA strategy bundles systems engineering tools into an iterative, five-step process applied at the facility-level to give frontline workers and managers a systems view of cascade performance, identify priority areas for improvement, discern modifiable barriers, and test workflow modifications and has been previously described in the literature (Fig. 1). Procedures for the adapted SAIA-MOUD’s five steps include:

#### Step 1: Understand MOUD performance and identify priority areas for improvement

**Fig. 2:**
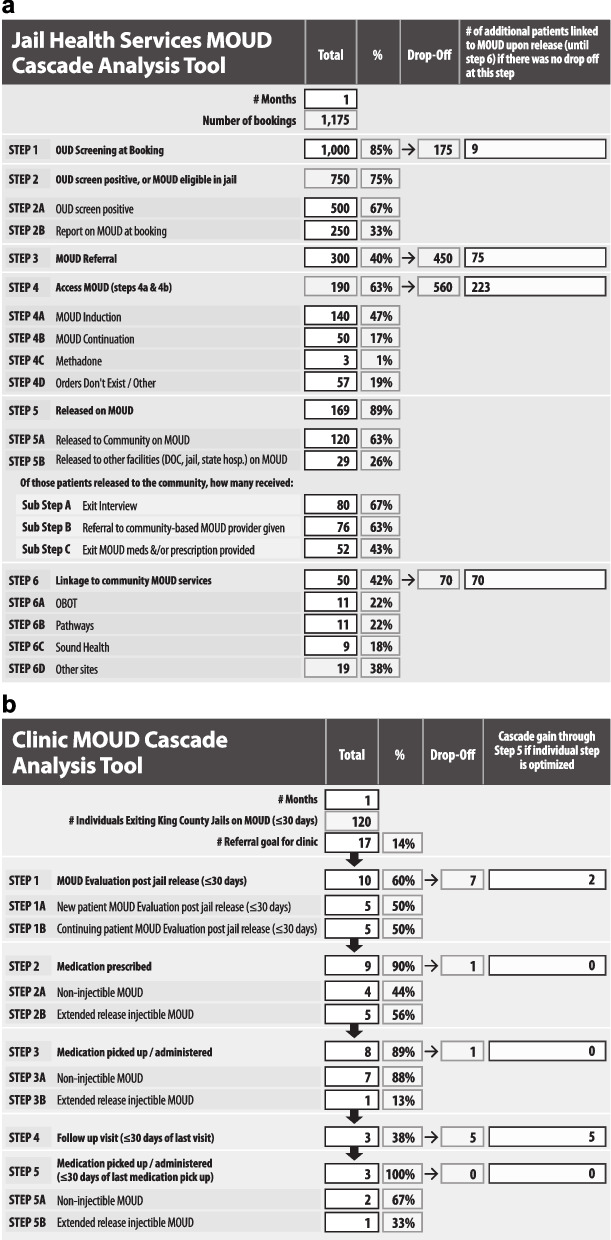
**a** JHS MOUD cascade analysis tool (MCAT). **B** Clinic MOUD cascade analysis tool (MCAT)

The MOUD Cascade Analysis Tool (MCAT) uses routine data to provide a rapid view of drop-offs along the MOUD cascade. Two MCATs have been developed and refined for this study. As an analytic tool, the jail-level MCAT provides care staff with a view of the greatest potential for flow improvements through OUD screening➔MOUD referral➔MOUD access➔release on MOUD as well as linkages between jail and MOUD referral clinics (Fig. 2a). Facilitative sub steps including client’s receipt of an exit interview, referral to community-based MOUD clinic, and/or MOUD prescription or carry medication, are captured but do not contribute to the cascade analysis calculation. The clinic-level MCAT offers health teams a view of the greatest potential for flow improvements for individuals exiting jail on MOUD within 30 days through MOUD evaluation➔ MOUD prescription➔ MOUD pick up/administration➔ follow up visit within 30 days of first visit➔ MOUD pick up/ administration (at second visit) (Fig. 2b).

#### Step 2: Identify facility-level modifiable bottlenecks using process mapping

**Fig. 3:**
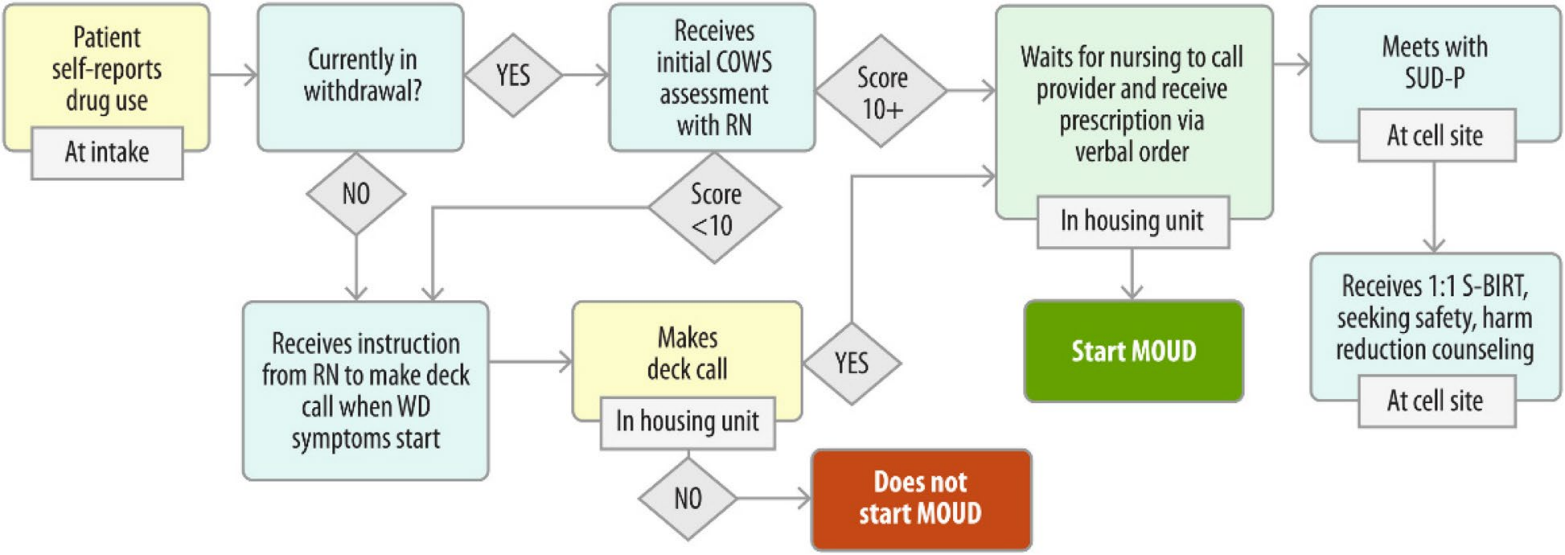
Ex, jail-based process map

Enabling facility staff to identify and gain consensus on specific bottlenecks to address in their MOUD care system is essential to defining innovations to implement. The SAIA applies sequential process flow mapping (Fig. 3.), [24] coupled with workflow observation, to identify bottlenecks and guide discussion on workflow modifications across MOUD services.

#### Step 3: Define and implement facility-specific workflow adaptations to address modifiable bottlenecks

After identifying modifiable barriers within cascade steps, facility staff select a specific change to improve performance. Selected workflow adaptations should be within the scope of influence of facility management and staff, feasible to implement, and expected to lead to rapid, substantial improvements in the targeted cascade step. Ideas for adaptations come from brainstorming solutions with facility-level staff, best practices from high performing MOUD services and published literature. Changes can be structural (e.g. re-purpose or modify consult rooms, alter staffing patterns), or process-oriented (e.g. modify visit schedules, engage partners in MOUD, enhance stigma reduction counseling). A workplan for the innovation (‘micro-intervention’) is developed by facility personnel to ensure consensus, delineate the MOUD cascade step and bottleneck addressed, and clarify operational design and roles.

#### Step 4: Monitor changes in performance and adopt / adapt / abandon

Facility staff monitor improvements in MCAT performance from the micro-intervention by measuring the increase in the proportion of patients progressing through targeted steps. Based on results, facility staff decide to adopt the change as standard practice, adapt and re-test the modification, or abandon it.

#### Step 5: Repeat cycle

Systems engineering process improvements are iterative, with ongoing testing of innovations responsive to evolving, contextually specific barriers. Facility staff repeat steps 1–4 at the end of each cycle, focusing on identifying new approaches to modify previously identified barriers, or if the first cycle was successful, testing micro-interventions to new bottlenecks identified in a repeated systems analysis.

### SAIA-MOUD trial design

Using a three-year quasi-experimental design, we will prospectively evaluate the effectiveness of SAIA-MOUD on improving MOUD care cascade quality and continuity for patients receiving care in jail and exiting to referral clinics in King County, WA (StaRI Checklist, Additional file 1) [36]. Clinic teams with support from the study team will deliver SAIA-MOUD at the jail-based MOUD program and three referral clinics over a two-year intensive phase, followed by a one-year sustainment phase where SAIA implementation will be led by JHS and MOUD clinic managers without study personnel support to enable pragmatic evaluation of sustained implementation (Table 1). The trial will culminate in a dissemination package, summarizing trial results and providing implementation and cost guidance to support state-level SAIA-MOUD scale-up. The mixed-methods evaluation will assess the impact of SAIA-MOUD on patient-level outcomes (Table 2).

**Table 1:**
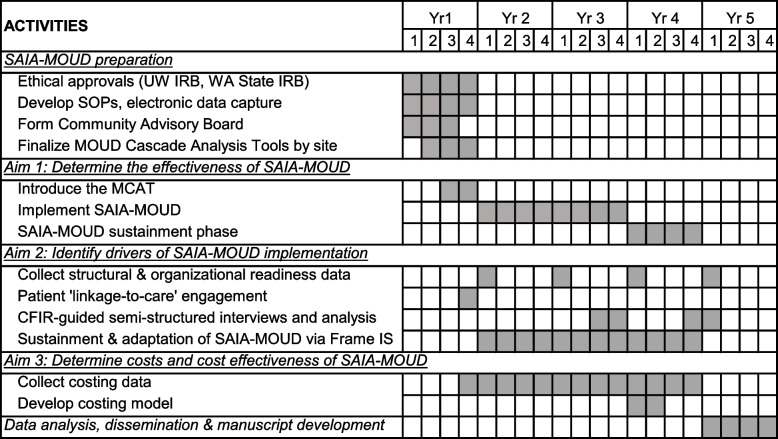
SAIA-MOUD study timeline

Key: SAIA-MOUD: Systems Analysis and Improvement Approach for the Medications for Opioid Use Disorder Care Cascade, *UW* University of Washington, *IRB* Institutional Review Board, *WA* Washington, *SOPs* Standard Operating Procedures, *MCAT* MOUD Cascade Analysis Tool

**Table 2:** SAIA-MOUD study outcomes

	**Outcome**	**Indicator**	**Numerator**	**Denominator**
Primary	Linkage	MOUD receipt ≤ 30 days of jail release	# of patients on MOUD in jail who upon release receive MOUD at a referral clinic ≤ 30 days	# of patients on MOUD in jail and released to the community
Secondary	Retention	MOUD receipt ≤ 30 days of initial linkage	# of patients on MOUD in jail who link to MOUD services upon release and subsequently return for a second receipt of MOUD ≤ 30 days	# of patients on MOUD in jail who upon release receive MOUD at a referral clinic ≤ 30 days

Covariates: sex, race, ethnicity, housing stability

### Process for introducing SAIA

SAIA-MOUD’s standard operating procedures (SOP) including the timing/duration, training schedules and intervention component guidelines (MCAT, process mapping and CQI) have been developed and acceptability tested with frontline MOUD staff and managers in the proposed study setting as part of formative work. In the first month of intervention, each facility team (including managers and staff from a range of sectors) will receive an on-site, 2-day orientation from the study team on the SAIA-MOUD SOPs. During this orientation to SAIA-MOUD implementation, facility teams jointly populate and interpret the MCAT, develop process maps of current patient care pathways to initiate or continue MOUD at their sites or referral clinics, and define 1–2 micro-interventions, and indicators to monitor the impact of these modifications.

In the first month of the SAIA-MOUD trial, each facility team will receive two 60-min supervision visits by study personnel, followed by monthly visits throughout the remainder of the 24-month intensive implementation period. During the 12-month sustainment period monthly mentorship visits will be conducted internally by the site team without support from study personnel to evaluate SAIA-MOUD sustainment with moderate resource investment.

Based on the other SAIA adaptations and the development process of the MCAT and its beta testing, we expect that analysis and improvement cycles will occur monthly, with an average of 12 cycles per year per implementing facility.

### Study setting

Our study will be conducted in King County, the most populous county in Washington State (WA), which reports rising rates of opioid-related overdose and high prevalence of OUD in carceral settings [21]. The SAIA-MOUD intervention will be introduced in four sites — 1) King County Jail Health Services (JHS) which is co-located across two jail-based clinics (King County Correctional Facility’s (KCCF) and Maleng Regional Justice Center’s (MRJC) MOUD program), Harborview Medical Center’s Opioid Treatment Network (HMC-OTN) (co-located across five clinic service points), Pathways Clinic, and Sound Health. With the exemption of the MRJC, which is in Kent, WA, all SAIA-implementing sites are based within walking or easy bus-ride distance of one another in central Seattle, where the majority of opioid overdoses occur in King County [1].

JHS has been a provider of MOUD since 2008, when methadone continuation was authorized. Ten years later, in June 2018, buprenorphine continuation was offered to inmates with existing prescriptions. In 2020, a pilot of buprenorphine initiation was conducted in JHS, and in 2021 it was integrated as part of a broader coordinated enhanced discharge program, making JHS an early-adopter of jail-based MOUD provision in the state [37]. JHS is one of the largest jails in Washington, housing ~ 1200 incarcerated people (approximately 10% of Washington’s jailed population) daily [38]. JHS provides MOUD services to ~ 150 patients on any given day; [37] however, gaps in the hand-off between jail and MOUD clinical services upon patients’ release contribute to unacceptable levels of opioid overdoses at this high risk “in-between” time.

HMC-OTN (a UW-affiliate), Pathways (a PHSKC-affiliate) and Sound Health (a community-based mental health non-profit organization) are similarly patient-centered, low barrier clinics, designed to be most accessible for marginalized populations, such as those exiting jail. All community-based clinics offer wrap-around services, including referrals to services that meet patient-identified, non-clinical needs, including housing, food and job-seeking supports. According to clinic managers each clinic sees ~ 150–350 MOUD patients monthly, including approximately 5–10% who are exiting JHS, and most staff have been employed for at least two years.

### SAIA-MOUD impact assessment

Through SAIA-MOUD’s mentored process of solution identification and testing, we hypothesize that its use will lead to rapid and sustainable improvements in MOUD service delivery and care continuity for individuals exiting jail and linking to MOUD care upon release.

#### Study population

All adults (≥ 18 years) accessing MOUD care services during the study period including those newly initiating or continuing MOUD treatment while incarcerated in King County jails and exiting to the community.

#### Exposure definition

Facilities will be considered unexposed prior to SAIA-MOUD intervention starting in their health facility and exposed thereafter. Individuals’ exposure to the intervention will be based on the exposure status of the facility in the calendar month in which they enter care (including those newly identified as eligible for MOUD and those already diagnosed with substance use disorder and on a medication for opioid use disorder).

#### Outcomes

In the main outcome – linkage to MOUD services within 30 days of release from jail (primary goal) – we will assess whether the proportion of clients who link to care (ie. receive MOUD at a referral clinic) within 30 days of release increases from the 24-month baseline period (January 2022 through December 2023) to the 24 months post-introduction of the intervention (Table 2). Linkage is defined as MOUD prescription filled or administered at the referral site. Rather than 14 days, 30 days was chosen as the review period of interest in order to capture individuals taking the recently introduced extended release injectable MOUD which are administered monthly. Since implementation preparation activities took place throughout 2024, this baseline period is excluded.

The secondary outcome analysis will assess retention in care within 30 days after initial linkage for patients in the three SAIA-MOUD clinics compared with retention in care within 30 days for all other patients linked to MOUD services in King County. Again, retention is defined as MOUD prescription filled or administered. This patient-level analysis will assess changes in the whether patients returned within 30 days after initial linkage (a binary yes/no variable) and only patients seen by providers seeing at least 10 patients in a year will be included.

Additional exploratory analyses will assess sustainment of our primary and secondary outcomes in the 12 months after our 24-month intensive implementation phase, as well as effect modification by salient client-level factors.

#### Data sources

Multiple routine data sources will be used to measure our effectiveness outcomes, which are routinely integrated into the Integrated Data Hub (IDH) which is housed jointly by Public Health—Seattle & King County (PHSKC) and King County’s Department of Community and Health Services. The IDH includes data from multiple sources of interest – including the Medicaid payer system (for which over 93% of those leaving JHS are eligible), jail booking data, and Homelessness Management Information System. With approval from the WA State Department of Health, both the Prescription Monitoring Program (PMP), and mortality data will be linked to the IDH data using a PHSKC machine learning approach, developed for another study. Data from the IDH will be cross-checked with clinic data reports generated through the EPIC system on a quarterly basis to assess data availability and completeness. Inconsistencies will be explored at the site level.

#### Power and sample size

Power estimates are for our primary analysis – to detect an impact of SAIA-MOUD on linkage to MOUD referral services within 30 days of release from JHS. Our primary analysis will be a difference in proportions test. Assuming a baseline proportion linked of 22%, and a conservative estimate of 100 people beginning or continuing MOUD in jail and releasing to the community each month (or 2,400 total releases in each of the baseline and intervention periods), we have 80% power to detect a change in the proportion linked of 3.3% percentage points or greater, and 90% power to detect a change in the proportion linked of 3.9% percentage points or greater. Power calculations were performed using the power.prop.test function in R.

#### Data analysis

For our primary linkage outcome we will measure the change in proportion of clients linked in two ways: 1) a difference in proportions test comparing the average share of clients linked in the baseline period to the average share of clients linked in the intervention period and 2) a non-parametric interrupted time series (ITS) analysis. For the ITS, the proportion of clients linked will be aggregated quarterly, and the model will be adjusted for covariates including sex, age, race, ethnicity and housing status at jail release, treatment experience prior to incarceration in jail and form of MOUD provided in jail.

For our secondary analysis on retention in care, patients initially linking to SAIA clinics will be matched to patients initially linking to non-SAIA clinics using propensity score matching. Variables used in the propensity score will include sex, age, race, ethnicity and housing status at jail release, treatment experience prior to incarceration in jail and form of MOUD provided in jail, in addition to the baseline period retention rate of the index provider seen following release. Our primary approach will rely on inverse probability weighting with the estimated propensity scores. We will also assess the robustness of our results using a kernel-density-based matching estimator using the propensity scores. Our estimate of interest is the average treatment effect on the treated (ATT).

### Exploring determinants of adoption, implementation and sustainment of SAIA-MOUD

Implementation outcomes—adoption, implementation, and sustainment—are important preconditions to ensuring attainment of desired service-level process and client-level health outcomes [39]. Though models of MOUD delivery in carceral and clinical settings are increasingly being designed and implemented, few studies are targeting development and testing of strategies that improve linkages between carceral and community settings of MOUD delivery. By describing to what extent SAIA-MOUD is adopted, implemented, and sustained, and identifying determinants (barriers and facilitators) of these implementation outcomes, we will endeavor to explain the linkage and retention in care results of this trial. Furthermore, results will inform the selection of core components and adaptations to SAIA-MOUD and serve as a common language to enhance transferability of findings to other contexts. Finally, we will document and describe adaptations to SAIA-MOUD to capture the dynamic process as the strategy evolves to better fit context.

#### Adoption

Adoption will be measured at the organizational level. For the purposes of this study, adoption will be defined as 1) identified staff from the target organizations (JHS, HMC-OTN, Pathways, Sound Health) attending SAIA-MOUD training, and 2) completing the first SAIA cycle. Based on previous SAIA trials and engagement with JHS, we expect high adoption of SAIA-MOUD (target: 80%). Describing determinants of adoption will provide actionable information to guide further intervention expansion. The Organizational Readiness for Implementing Change (ORIC) scale will be used to elucidate drivers of readiness to adopt SAIA-MOUD at the facility level, which will also be explored via qualitative evaluation of SAIA-MOUD guided by the Consolidated Framework for Implementation Research (CFIR).

*Organizational Readiness for Change* refers to the extent to which organizational members are psychologically and behaviorally prepared to implement organizational change, which affects decisions to adopt interventions like SAIA-MOUD [40]. ORIC is a 12-item Likert-type scale, broken into domains of change commitment and change efficacy that we have employed effectively in other SAIA trials to capture the extent to which organizational members are psychologically and behaviorally prepared to adopt and implement organizational change [27, 41]. The ORIC will be administered after the second month of SAIA-MOUD meetings to two managers and six frontline staff per organization (*n* = 32). Analysis will test whether sufficient inter-rater reliability and inter-rater agreement exist to aggregate individual responses to the organizational level [42–45]. If tests do not justify aggregation, we will use a measure of intra-organization variability in readiness rather than an organization-level mean in our analysis [43, 45]. The resulting analysis will provide readiness profiles for each organization as they initiate implementation, which will complement adoption, implementation, and effectiveness data in understanding the impact of SAIA-MOUD.

#### Implementation

Implementation fidelity is measured at the organizational level and will be assessed monthly using the SAIA monitoring system. Subsequently, implementation determinants will be explored via CFIR-guided qualitative inquiry with staff in both jail and referral MOUD clinics.

##### Implementation fidelity

A monthly implementation monitoring system will prospectively capture whether the individual SAIA-MOUD components (cascade analysis, process mapping and CQI cycles) were implemented each month, allowing us to describe implementation dose throughout the study [17]. Study team members will enter this information into a tablet-based RedCAP fidelity monitoring tool monthly (Table 3). Tracking measures of fidelity will provide an indication of SAIA-MOUD core components (*versus* peripheral/modifiable). Changes in fidelity patterns of over time in each of the SAIA-MOUD sites will be further explored via qualitative inquiry.

**Table 3:** SAIA-MOUD fidelity monitoring tool

**Fidelity: **Was a SAIA cycle conducted with fidelity that month? (All must be YES)
Monthly SAIA meeting occurred (yes/no)
Workplan developed (yes/no)
Workplan was implemented fully or partially (yes/no)
**Fidelity with Quality: **Were workplans developed with quality? (All must be YES)
Micro-intervention is an appropriate solution for the problem (yes/no)
Micro-intervention is appropriate for the targeted MOUD cascade step (yes/no)
Micro-intervention is a clearly delineated task(s) assigned to a specific person/people (yes/no)

Micro-intervention is not an exact (verbatim) repeat of the previous month's past micro-intervention at the facility (yes/no)

##### Qualitative data collection and analysis

In-Depth Interviews (IDIs) and Focus Group Discussions (FGD) will be held with organizational staff in the final quarter of the intensive implementation period to examine the implementation process, define core SAIA-MOUD components, and describe determinants of success and failure in implementing organizations. A total of two staff in each implementing organization (eight total) will be interviewed, and four FGDs (one per organization) will be held. The CFIR, an established determinants framework, is well suited to this sub-aim, and will be used to develop interview and discussion guides to assess the multilevel factors that influence intervention implementation and effectiveness [46]. Interview and discussion guides will be developed using questions from the CFIR wiki guide to address selected constructs from the five CFIR domains. IDI and FGD guides will include questions adapted from the CFIR question bank to address the selected CFIR constructs (Table 4); the topics covered in each type of data collection will not differ, as we are interested in understanding group norms about each topic (via FGDs) and noting whether there are minority opinions (identified via IDIs). FGDs and IDIs will be conducted by an experienced facilitator (FGDs will be accompanied by a note-taker), audio-recorded, transcribed.

**Table 4:** Ex. CFIR-guided questions

**Outer Setting**
***External Policies & Incentives***
What kind of county, state or national performance measures, policies, regulations or guidelines influenced the decision to implement SAIA-MOUD?
**Inner Setting**
***Structural***
What kinds of infrastructure changes are needed to accommodate SAIA-MOUD? Changes in scope of practice? Changes in formal policies? Changes in information systems or electronic record systems? What kind of approvals are needed?
**Process**
***Engaging—Opinion Leaders***
Who are the key influential individuals that need to be engaged with SAIA-MOUD implementation to make it work? What are influential individuals saying about the SAIA-MOUD?

FGDs will range in size from 7–10 participants, which is sufficient to generate conversation without being too large to become intimidating [47]. We will conduct IDIs with managers at each organization. By purposively holding FGDs for frontline staff separately from manager IDIs, we aim to identify opinions that lower ranking staff may feel uncomfortable sharing with their superiors, or issues related to staffing that higher ranking staff feel uncomfortable discussing with subordinates, which may be salient determinants of successful implementation in each site. The IDIs with managers will allow for exploration of the individual experience with the SAIA-MOUD, and reflection on adaptations experienced over the intensive intervention period. We expect that two IDIs and one FGD per organization will ensure > 80% of the staff at each site will be involved in providing input.

##### CFIR-guided analysis

Thematic analysis of qualitative data will follow CFIR domains and constructs to distinguish content and structure of the SAIA-MOUD training, materials, and mentorship *vs.* how SAIA-MOUD was received and implemented at the site. A two-step process will be used for analysis to identify drivers of implementation success and generate an in-depth understanding of implementation processes and predictors. First, two coders in a stepwise, iterative fashion will code the IDI and FGD transcripts and conduct content analysis within a deductive framework to identify key implementation themes (using selected CFIR constructs but allowing flexibility for other themes to emerge). Coding will be compared across pairs and differences discussed prior to final coding. Second, case memos will be written, and three analysts will assign ratings for each construct. Using a rating process previously applied to the CFIR, [48, 49] ratings will reflect the positive or negative influence (valence) and the strength of each construct. Constructs will be coded as missing too much data (M), not (0), weakly (+ 1/-1), or strongly (+ 2/-2) distinguishing low/high performance. Findings will inform recommendations for SAIA-MOUD, including identifying intervention core components, explaining intervention adaptation, and documenting lessons learned.

#### Sustainment

Moore et. al. defines sustainment as, “[a]fter a defined period of time, the program, clinical intervention, and/or implementation strategies continue to be delivered…while continuing to produce benefits for individuals/systems” [50]. Sustainment is a key construct of implementation science, and the field has called for an expanded research focus that moves beyond describing barriers to developing an evidence base on strategies to address barriers [39, 51]. We will describe sustained implementation of SAIA-MOUD and assess determinants of sustainment at each site using similar procedures described for the implementation period throughout the 12-month sustainment (non-intensive) period, including 1) describing fidelity to the monthly SAIA-MOUD protocol, paired with 2) qualitative inquiry (IDIs and FGDs) with site managers and frontline staff (using the same sampling, data collection and analysis techniques described above). Findings on sustained implementation of SAIA-MOUD without intensive support from research staff will complement initial effectiveness findings on client outcomes.

#### Describing adaptations

Adaptations to SAIA-MOUD will be prospectively documented during both the intensive and maintenance phases using the FRAME-IS, a framework developed to document modification to implementation strategies [52]. FRAME-IS is designed to monitor the component of the strategy adapted (content, training, context, etc.), the nature of the modification (tailoring, packaging, adding or removing elements, etc.), the goal of the adaptations (increase reach, adoption, acceptability, sustainability, etc.), and the level of the adaptation (organizational, implementer, clinician, etc.) By documenting adaptations and their motivations, FRAME-IS supports determining the processes or mechanisms through which implementation strategies influence implementation outcomes. We will incorporate FRAME-IS into the REDCap-based fidelity monitoring tool that is filled out monthly via tablet by those leading the SAIA cycles. There will be a checkbox to indicate if – during the month in question – there were any changes to the core SAIA-MOUD components (e.g. was cascade analysis, process mapping or quality improvement not conducted), and if so, the FRAME-IS questions will open to describe the goal, nature and content of these changes. At the end of the trial, data will be used to describe adaptations observed during both the intensive and non-intensive (maintenance) phases of the study.

### Estimating the cost and cost-effectiveness of SAIA-MOUD on improving equitable access within carceral and community settings, including costs of linkage across services

We will evaluate the cost-effectiveness of SAIA-MOUD from the county government, societal and healthcare sector perspectives.

#### Cost-effectiveness analyses

We will conduct both a cost-effectiveness analysis (incremental cost per additional person linked to care) and a cost-utility analysis (incremental cost per Quality-adjusted life-year (QALY) gained). Comparative effects between SAIA-MOUD and *status quo* will be estimated from our primary outcome of linkage to MOUD services post release from jail. Analyses will be conducted from the county, healthcare sector, and societal perspectives, and costs included in each perspective (Table 5).

**Table 5:** Impact inventory of outcomes

Outcome	Included in Perspectives	Data Source	Analysis notes
SAIA-MOUD intervention	All	Time-motion studies	Micro-costing (see below)
Healthcare utilization and costs	All	Medicaid-data	Regression analysis
OUD-related	All	Medicaid-data	Regression analysis
OUD-unrelated	All	Medicaid-data	Regression analysis
Out-of Pocket	All	Medicaid-data	Regression analysis
Shelter/Respite Care	Soc, State	State Homeless Management Information Systems	Use unit cost approach
Criminal Justice Impact of Recidivism	Soc, State	State Criminal justice system	Direct costs of criminal activity to state
Societal Impact of Recidivism	Soc	Literature-based estimate	e.g., Property damage, pain, and suffering, etc
Net Productivity	Soc	No direct source	Will remain unmeasured
Welfare payments	State	State databases	Food and cash assistance
Life years	All	State Death Registry	1-Year survival analysis
Quality-adjusted life years	All	Medicaid-data	Quality of life weight based on diagnosed conditions in Medicaid

Costs for SAIA-MOUD will be measured using time-driven activity-based costing, [53] which assigns costs to personnel time required to complete implementation procedures. Procedures will be identified from the SAIA specification, [24] and time to complete these procedures will be gathered using a combination of participant self-report and supervisor report. Wage rates and overheads will be gathered from interviews with finance personnel. Costing will include all start-up and recurrent activities and measure resource use and costs from intervention design through sustainment at the clinics. Total costs will be divided by the total number of persons exposed to study sites implementing SAIA-MOUD to obtain an estimate of SAIA-MOUD cost per person.

Health state utilities used in cost-utility analysis will be estimated from diagnosed conditions in Medicaid data and from the published literature. We will calculate incremental cost effectiveness ratios (ICERs) by dividing the difference in costs between SAIA and *status quo* by the difference in outcomes. Uncertainty in estimates will be obtained via 1000 bootstrapped deviates. Cost-effectiveness acceptability curves will be used to communicate uncertainty to policymakers. Subgroup analysis will be carried out based on first-year versus second-year of SAIA-MOUD implementation, as well as covariates of interest (sex, race, ethnicity and housing stability).

#### Power

No power calculations are provided as Aim 3 procedures will rely on analyses articulated in Aim 1, together with cost data. As noted, uncertainty in estimates will be obtained via 1000 bootstrapped deviates.

### Trial status

Preparations for SAIA-MOUD initiated in January 2024. Initiation of SAIA-MOUD trial is planned for January 2025.

## Discussion

In this trial we will assess SAIA-MOUD, a novel and flexible implementation strategy to improve OUD screening, treatment and linkage to care for people exiting carceral settings. SAIA uses systems engineering tools to visualize care cascades, quantify gaps in care, and center patients’ experiences of care in quality improvement efforts. This approach allows health workers to prioritize interventions that can move patients more efficiently and equitably through critical care steps. SAIA is flexible and adaptable to local settings, empowering health care workers to test their ideas for optimization through a series of “micro-changes”. Rather than testing a single intervention that may become irrelevant after policy or technology changes, SAIA has longevity, as the approach is adaptable to the changing service landscape, which increasingly targets conditions like OUD.

We will employ innovative and robust implementation science methods to evaluate SAIA-MOUD. While more jails are adopting MOUD, much remains to be learned about addressing implementation challenges related to expansion of MOUD in carceral settings and linkage to clinical care upon re-entry [54]. Our design represents a novel application of SAIA because it will be implemented across levels of the health services, jail-based and community-based, with the goal of optimizing linkage to care after release, and ultimately decreasing opioid overdose in the vulnerable post-release period. Our design includes a range of implementation science methods including the ORIC scale [40] to assess facility readiness for SAIA-MOUD adoption, the CFIR to guide intervention planning, implementation, and address “what works, where and why”, through identification of implementation determinants to support further SAIA-MOUD implementation across diverse settings [46]. Adaptions to the SAIA-MOUD implementation strategy will be tracked throughout intensive and sustainment phases via FRAME-IS [52]. Additionally, cost effectiveness analyses rarely assess MOUD management strategies for individuals involved in the criminal legal system. This study will use a micro-costing approach to estimate the incremental cost per additional patient passing through the MOUD cascade from jail to clinical referral services. State of the art implementation science methods strengthen our evaluative framework for multi-level, theory-based adaptation of interventions.

## Supplementary Information

Additional file 1: StaRI checklist.

## Data Availability

There are multiple data sharing agreements (DSAs) that support this research study, in particular related to the effectiveness and costing aims, and a standard requirement across these DSAs is to destroy research data sets at the completion of the study. The implementation related datasets collected/used and/or analyzed during the current study are available from the corresponding author on reasonable request.

## Abbreviations

ATT: Average Treatment Effect of the Treated
CFIR: Consolidated Framework for Implementation Research
CQI: Continuous Quality Improvement
DAJD: Department of Adult and Juvenile Detention
FGD: Focus Group Discussion
FRAME-IS: Framework for Reporting Adaptations and Modifications to Evidence-based Implementation Strategies
HMC-OTN: Harborview Medical Center’s Opioid Treatment Network
IDH: Integrated Data Hub
IDI: In-Depth Interview
ITS: Interrupted Time Series
JHS: Jail Health Services
KCCF: King County Correctional Facility
MCAT: MOUD Cascade Analysis Tool
MOUD: Medications for Opioid Use Disorder
MRJC: Maleng Regional Justice Center
ORIC: Organizational Readiness for Implementing Change
OUD: Opioid Use Disorder
PHSKC: Public Health—Seattle & King County
PMP: Prescription Monitoring Program
QALY: Quality-Adjusted Life Year
SAIA: Systems Analysis and Improvement Approach
SOP: Standard Operating Procedures
WA: Washington State
